# Evaluating an Algorithm for Prescribing Mildly Thick Liquids as a Temporary Bridge to an Oral Diet in the Acutely Hospitalized Population

**DOI:** 10.7759/cureus.112371

**Published:** 2026-07-09

**Authors:** Melodyanne Cheng, Sarah Panjwani, Jennifer L Long

**Affiliations:** 1 Otolaryngology - Head and Neck Surgery, University of California Los Angeles David Geffen School of Medicine, Los Angeles, USA; 2 Otolaryngology - Head and Neck Surgery, University of California Los Angeles, Los Angeles, USA; 3 Surgery and Perioperative Medicine, Veterans Administration Greater Los Angeles Healthcare System, Los Angeles, USA

**Keywords:** algorithm, dysphagia, mildly thickened liquids, patient safety, swallow study

## Abstract

Objective

This study aims to evaluate the safety of the temporary use of mildly thick liquids (MTL) prescribed at bedside using a stringent algorithm in hospitalized patients.

Study design

This retrospective chart review study included hospitalized patients who had a consultation with speech pathology for clinical swallow evaluation and were given diet recommendations of MTL. Exclusion criteria involved patients who were initially seen for an instrumental assessment (not at bedside) and patients who did not receive speech-language pathologist (SLP) follow-up (due to a change in medical status, diet managed by the medical team after initial assessment, etc.)

Methods

Data from bedside SLP evaluation or instrumental swallow evaluation (modified barium swallow study (MBSS) or fiberoptic endoscopic evaluation of swallowing (FEES)) during hospitalization were collected. Radiologic chest imaging reports from before and after MTL diets were reviewed for possible aspiration or aspiration pneumonia. Descriptive statistical analysis was conducted using Excel.

Results

Of 1000 charts reviewed, 162 patients met the inclusion and exclusion criteria. Seven of these 162 patients (4.3%) had radiologic findings suggesting concern for aspiration after the MTL diet was prescribed. Six of these patients had medical factors that contributed to the radiologic finding; thus, a direct relationship between MTL diet and concern for aspiration could not be established.

Conclusions

This study supports consideration of temporary MTL prescribed at bedside with a stringent algorithm, given the small number (4.3%) of patients with aspiration concerns after MTL diet on follow-up. This approach may shorten the time to oral diet and hospital discharge in carefully selected patients.

## Introduction

Recognized by the World Health Organization, oropharyngeal dysphagia is defined as the inability to safely and effectively transfer a bolus of solids or liquids from the oral cavity to the esophagus [1]. Oropharyngeal dysphagia is a serious concern among hospitalized patients due to its impact on the safety and efficiency of oral intake, with a potential for severe consequences, including aspiration, malnutrition, and prolonged hospitalization [2]. In the in-patient hospital setting, mildly thick liquids (MTL), also known as nectar-thick liquids, have been utilized to reduce the risk of oropharyngeal aspiration since the 1990s, classically when transitioning patients from a nil per os (NPO) diet to a regular texture liquids (thin liquids) diet [3]. In comparison to thin liquids (1-50 centiPoise), MTL liquids (51-350 centiPoise) are more viscous and can improve swallow safety for patients with poor oral bolus organization or delayed pharyngeal swallow initiation [4-6].

The hospitalized patient often has limited nutrition/hydration options if there is concern for aspiration and resulting pulmonary complications with thin liquids. If a patient is deemed safe from aspiration risk with mildly thick liquid, this option is often preferred over enteral nutrition or nothing by mouth, as prolonged nothing by mouth status places the patient at high risk for malnutrition, prolonged hospitalization, and minimizes options for medication delivery [7]. A modified diet including mildly thick liquid may be preferred by the medical team and the patient when there is an expectation to improve in the short term (less than one week) or a need for optimization (following acute neurological injury, following a short intubation period, etc.) prior to instrumental swallow evaluation. However, research indicates that MTLs are not without risk. Studies looking at the effects of aspiration of MTL in animal models have raised concerns for initiating this diet at bedside without an instrumental swallow evaluation [8-9]. Long-term use of MTLs poses concern for dehydration, unsatisfied feelings of thirst, slow gastric emptying, risk of reflux, risk of pharyngeal residue, risk of aspiration without overt coughing, and other potential negatives [10]. At this academic tertiary hospital center, patients who demonstrate overt signs of aspiration with thin liquids are considered for temporary use of MTLs as a bridge to baseline diet using a strict algorithm. This study aims to evaluate the safety of this algorithm.

## Materials and methods

Study participants

This study was approved by the Institutional Review Board of UCLA and conforms to the tenets of the Declaration of Helsinki. A hospital electronic medical records (EMR) request was placed to identify all patients for whom MTLs were recommended by a speech-language pathologist (SLP) between December 2021 and December 2023. The search yielded over 1000 medical records. Of these, patients who received consultation to SLP for bedside swallow evaluation, documented diet recommendation of MTLs by SLP, and follow-up evaluation by SLP were included in the study. Exclusion criteria involved patients who were seen for instrumental swallow evaluation only and those who did not receive a follow-up by SLP due to a change in medical status, diet managed by the medical team following SLP assessment, etc. The final data set included 162 patients, on whom a retrospective chart review was conducted.

MTL algorithm for patient eligibility

**Figure 1:**
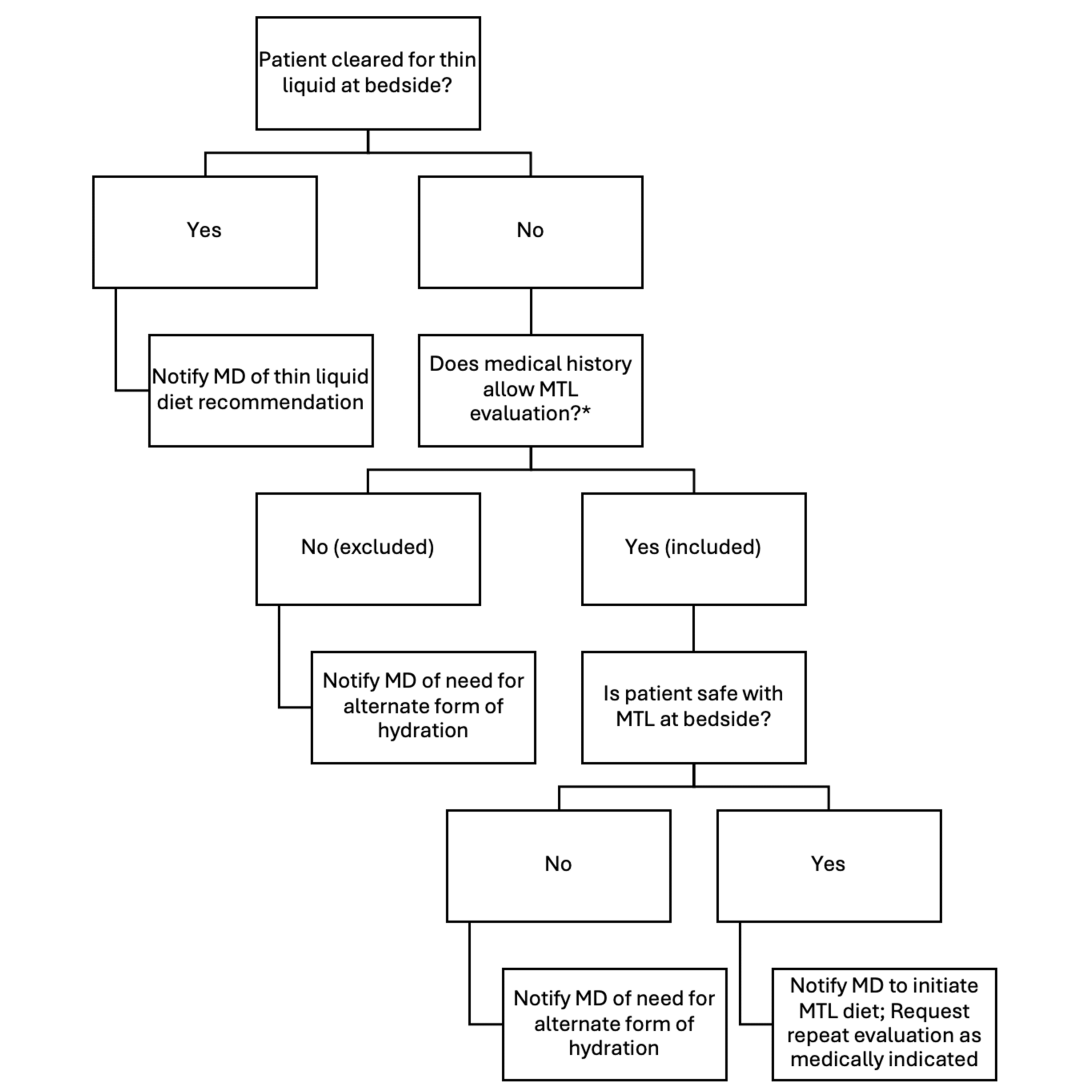
Algorithm for assessing patients at bedside for mildly thick liquid diet recommendations *refers to: dysphagia history, transplant history, aphonia, dysphonia, G-tube with no clear reasoning, history of aspiration with no overt signs MTL - mildly thick liquids

All patients who demonstrated overt signs of aspiration with thin liquids at bedside were evaluated by SLPs for a mildly thick liquid diet using the algorithm in Figure 1. This algorithm was created at the institution using evidence-based research methodology to define clinical populations not suitable for the MTL trial at bedside. Patients whose medical history places them at high risk for aspiration are excluded from MTL assessment at bedside. This includes patients with a history of oropharyngeal dysphagia, lung/liver transplant or acute cardiac surgery, and aphonia or severe dysphonia due to concern for aspiration without overt signs (cough, wet voice, etc.) [11]. Patients with a history of liver transplant are found to be at high risk for post-surgical oropharyngeal dysphagia due to mental status changes, prolonged intubation, and possible mechanical ventilation, and thus, out of caution, these patients were excluded [12]. Lung transplant patients are also at high risk for oropharyngeal dysphagia following intubation and due to "the vulnerability of the intrathoracic vagal nerve and its branches to injury during surgery," which may result in aspiration without overt signs (i.e., cough, wet voice, etc.) [13]. Research indicates that bedside swallow assessment, specifically a 3-ounce water swallowing test, has low sensitivity for the detection of aspiration in patients who are post-cardiac surgery [14]; thus, these patients are also excluded from bedside MTL assessment for the purposes of the algorithm (Figure 1).

Additionally, patients with a recurrent history of aspiration/aspiration pneumonia not related to a single isolated event (i.e., vomiting or acute illness) are excluded from assessment of MTL at bedside due to concern for unknown risk of oropharyngeal dysphagia. Patients with a medical history indicating high risk for sensory deficits (i.e., radiation to the larynx) are also excluded due to concern for poor sensory response to aspiration at bedside. Patients with a gastrostomy tube with no clear medical reasoning are also excluded due to the unknown risk for aspiration. The patients included by the algorithm are expected to improve in the short term (i.e., acute cerebral infarct, acute UTI, etc.) and have minimal factors for aspiration pneumonia (i.e., do not have COPD, were not frail, etc.), and therefore are ideal candidates for temporary use of MTLs. Patients in a palliative “comfort care” status who consented to MTLs were also included to provide them with comprehensive dietary options. Patients who were admitted after Trans Robotic Oral Surgery (TORS) were also included despite post-operative sensory deficits and the need for frequent pain medication. They were included because recent research indicates "early initiation of oral intake is not associated with an increase in postoperative complications", excessive coughing with thin liquid may irritate the post-op tissue, and there is reduced clinical concern for pulmonary complications given that these patients are often otherwise robust [15-16].

**Figure 2:**
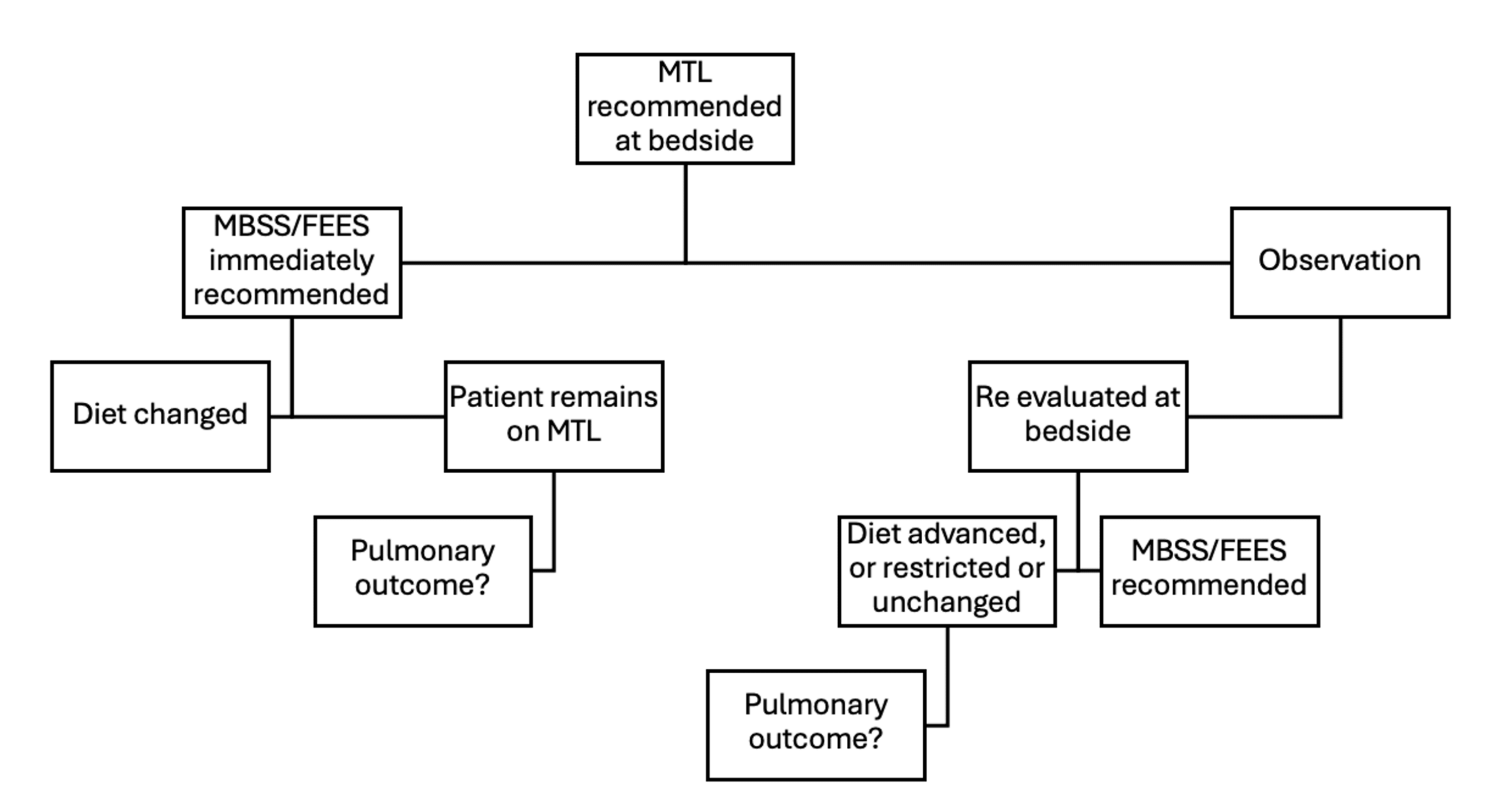
SLP follow-up algorithm for patients prescribed mildly thick liquid diet methodology MTL - mildly thick liquids; MBSS - modified barium swallow study; FEES - fiberoptic endoscopic evaluation of swallow

All patients prescribed MTL by the eligibility algorithm were then assessed by SLPs using 4 or more ounces (oz) of MTL at bedside. A diet with MTLs was only recommended if patients demonstrated no overt signs or symptoms of aspiration (wet voice, throat clear, cough, etc.). Following the prescription of MTLs, patients were seen for follow-up evaluation to assess readiness for diet advancement (Figure 2). Follow-up assessment was completed either at bedside (observation) or via instrumental evaluation (modified barium swallow study (MBSS) or fiberoptic endoscopic evaluation of swallow (FEES)) based on SLP clinical judgment [17-18]. Figure 2 depicts the algorithm used for follow-up assessment of patients prescribed MTL during admission.

Basic biographical information (age and sex), incident of a witnessed swallowing concern, and history of head and neck cancer was collected for all included patients. SLP documentation for each patient was thoroughly reviewed to log the initial bedside evaluation, all follow-up assessments, including repeat bedside evaluations and instrumental swallow evaluations, including modified barium swallow study (MBSS) or fiberoptic endoscopic evaluation of swallowing (FEES) during hospitalization. Imaging (chest X-rays, CT chest, CT thorax/upper abdomen) was also reviewed within a seven-day timeframe before and after patients were prescribed mildly thick liquid diets. Radiologic reads were reviewed for clinical language for “aspiration” or “aspiration pneumonia”. Descriptive statistical analysis was conducted using Excel.

International Diet Standardization Initiative (IDDSI) level 2 mildly thick consistency (also known as nectar thick liquid) was utilized during the time period of this retrospective study (IDDSI 2024). A gel-based xanthan gum thickener drink was prepared by SLP for all assessments. Once a mildly thick liquid diet was initiated, patients either drank a pre-thickened mildly thick liquid juice or a gel-based xanthan gum thickener was mixed into their drink. The choice of thickener type was based on patient preference, dietary constraints, and material availability. Those patients receiving xanthan thickener were served packets on all meal trays, and their nurse provided assistance mixing the thickener into drinks.

## Results

Demographics

In total, 162 patients were consulted to SLP for bedside swallow evaluation and met the inclusion/exclusion criteria. In terms of demographics of the overall patient cohort in this study, the mean age (+/- SD) was 74 (+/- 18) years, 65 (40.1 %) were female, and 97 (59.9%) were male. 33 patients (11.3%) had a witnessed swallowing concern reported by hospital staff, family, or the patient themself. Nine patients (3.1%) had a history of head and neck cancer.

**Table 1:** Demographics and clinical characteristics of hospitalized patients prescribed MTL MTL - mildly thick liquid

Demographic variable	n
Total number of patients	162
Average age in years (±SD)	74 (±18)
Gender	
Male	97 (59.9%)
Female	65 (40.1%)
By age in years	Number of patients
<20	2
20-29	2
30-39	5
40-49	9
50-59	9
60-69	22
70-79	39
80-89	45
90-99	26
>99	3
Clinical history	
Witnessed swallowing concern	33 (11.3%)
History of head and neck cancer	9 (3.1%)

**Figure 3:**
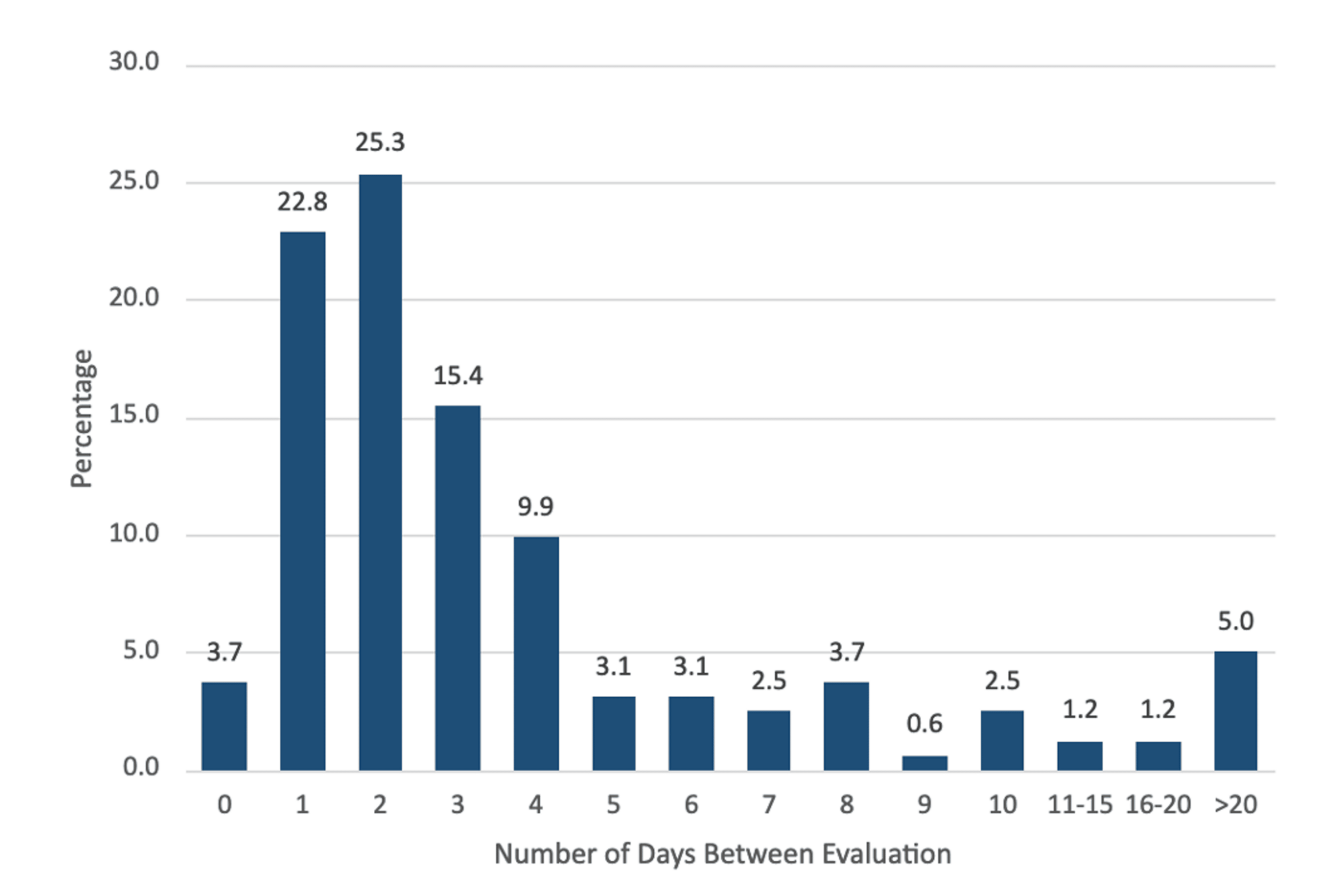
Distribution of days between initial evaluation and repeat evaluation

The number of days between the initial bedside swallow evaluation and follow-up repeat evaluation (bedside or instrumental) is outlined in Figure 3.

Instrumental versus bedside swallow evaluation

**Figure 4:**
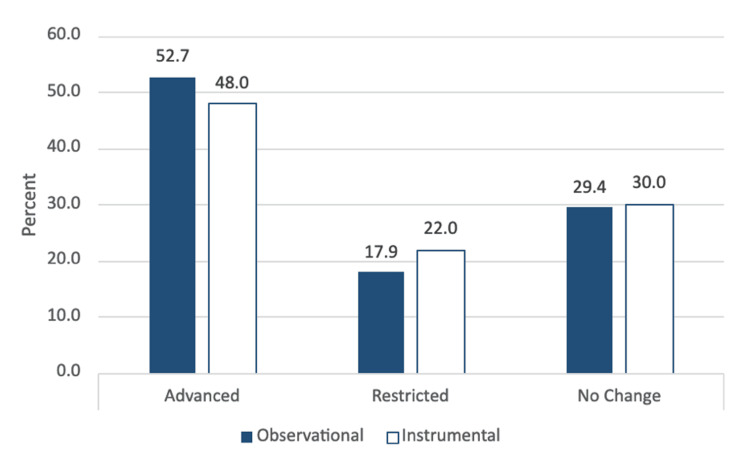
Follow-up swallow evaluation diet outcomes within observational and instrumental groups

50 patients (30.9%) underwent an instrumental swallow evaluation for the follow-up visit, and 112 patients (69.1%) underwent a bedside swallow evaluation at follow-up. Among the patients who underwent an instrumental swallow evaluation for follow-up, 24 patients (48.0%) had diets advanced to thin liquids, 11 patients (22.0%) had diets restricted (i.e., NPO), and 15 patients (30.0%) remained on mildly thick liquids. Among the patients who underwent a bedside swallow evaluation at follow-up, 59 patients (52.7%) had diets advanced to thin liquids, 20 patients (17.9%) had diets restricted (i.e., NPO), and 33 patients (29.4%) remained on mildly thick liquids. Figure 4details the percentage of patients whose diets were advanced, restricted, or unchanged during the follow-up evaluation. There was no statistically significant difference between the distribution of patients who had their diets advanced, restricted, or not changed in the instrumental group versus the observational group (p >0.05).

Radiologic imaging findings of patients on a temporary MTL diet

The American College of Radiology (ACR) recommends chest radiography as the initial imaging modality for patients at risk for aspiration pneumonia [19]. Radiologic imaging reports were reviewed for the terms "aspiration" and "aspiration pneumonia" to determine patient safety with the temporary MTL diet for all 162 patients. 106 (65.4%) did not have these terms in their imaging reports. 34 (21.0%) patients had the terms "aspiration" and/or "aspiration pneumonia" on imaging reports prior to MTL diet prescription. 15 (9.3%) patients had these terms on imaging reports both before and after temporary use of mildly thickened liquids. Seven (4.3%) patients were found to have new concern for aspiration (terms "aspiration" and "aspiration pneumonia" on imaging reports) only after the MTL diet was prescribed. The charts for all seven patients were reviewed in detail to determine if findings were related to the MTL diet. Two of the seven patients had these terms on new imaging after they were found to be safe for MTLs at bedside and instrumental assessment. One of the seven patients had these terms on imaging a few days after diet advancement to thin liquid via instrumental evaluation.

**Figure 5:**
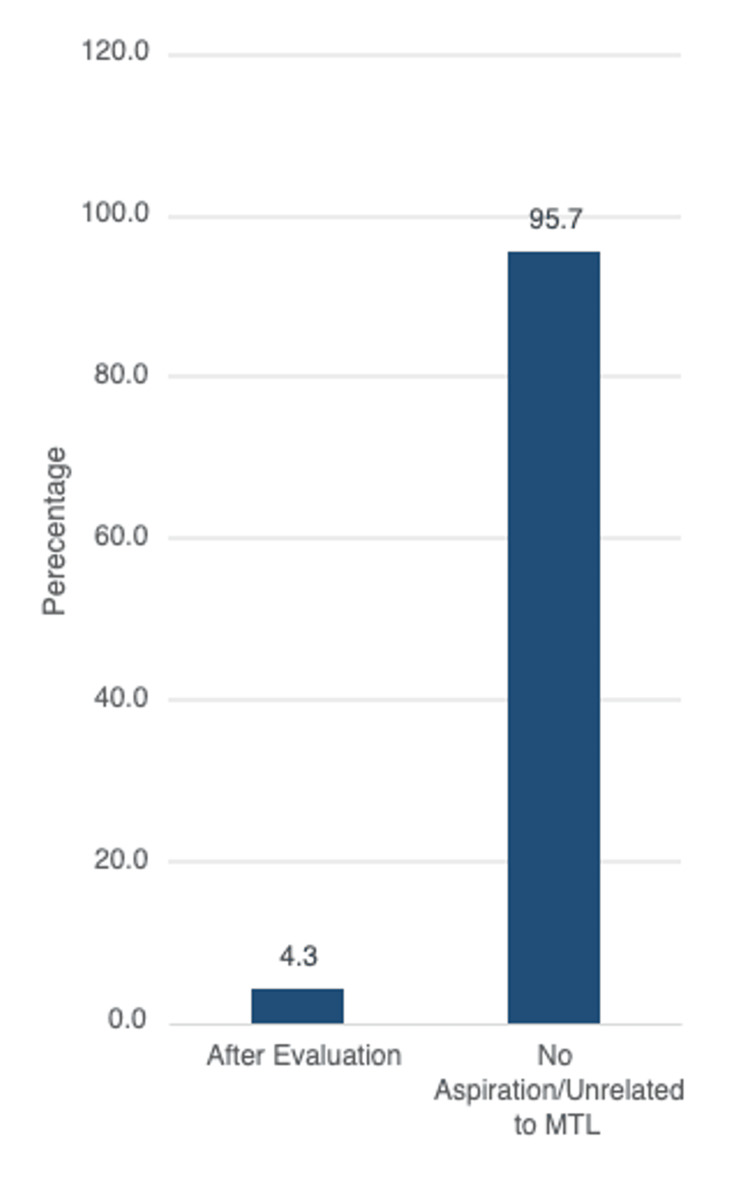
Aspiration findings on radiologic imaging (chest x-ray, CT chest, CT thorax/upper abdomen) among 162 patients who were prescribed MTL at bedside evaluation and received an SLP follow up evaluation MTL - mildly thick liquid; SLP - speech-language pathologist

Of the seven patients whose radiologic imaging indicated concern for aspiration after MTL diet initiation, further review of records revealed that two (28.6%) had imaging taken within one day after the initial SLP bedside evaluation (four hours and 15 hours). In addition, one (14.3%) patient had a pre-existing condition of aspiration pneumonia on hospital admission prior to MTL diet initiation, and five (71.4%) patients were severely sick with either acute changes in mental status, recorded episodes of productive nausea and vomiting, or loss of consciousness episodes after initial admission as possible confounding factors unrelated to MTL diet initiation for the patients demonstrating concern for aspiration on radiologic imaging. The median days between SLP evaluation and radiographic imaging was three days. Table 2 below provides additional medical details for these seven patients.

**Table 2:** Detailed medical conditions and time from SLP initial evaluation to imaging in the seven patients with concern for aspiration after MTL diet initiation SLP - speech-language pathologist; MLT - mildly thick liquid; MSSA - Meticillin-sensitive *Staphylococcus aureus*

ID	Complications during the hospital course	Days from SLP evaluation to imaging
1	Worsening mental status, nausea, vomiting, and decline in functional status 2/2 med dose change	3
2	Right facial droop and dysarthria; monitoring of focal motor seizures of right-side face concerning for Todd's paralysis	1 (4 hours)
3	Sepsis, MSSA Bacteremia 2/2 acute on chronic worsening back pain and new-onset dysarthria	7
4	Anoxic brain injury, intermittent delirium, intentional fentanyl overdose	5
5	Found to be COVID positive, started on antibiotics	4
6	Toxic metabolic encephalopathy, acute onset diarrhea, hyponatremia, deconditioning	1 (15 hours)
7	Admitted due to sepsis 2/2 aspiration pneumonia	2

Statistical analysis

All statistical analyses were performed using Excel (Microsoft, Redmond, Washington). Descriptive statistics (e.g., means with standard deviation; percentages of total) were used to summarize and report the demographic and clinical characteristics of the patients.

## Discussion

Diet modification to minimize aspiration risk is a common and highly impactful clinical care decision. Patients at risk for oropharyngeal aspiration of thin liquids due to medical conditions that are expected to improve in the short term can often continue oral liquids with modification to MTL consistency. In this study, we analyzed the baseline characteristics and outcomes of hospitalized patients with dysphagia who were temporarily prescribed mildly thick liquids via bedside evaluation using a stringent algorithm. We found that among patients prescribed MTLs at bedside, new aspiration noted on imaging was rare (only 4.3%). Overall results from this single institution suggest MTL can be prescribed at the bedside safely with a stringent algorithm.

The MTL diet was originally developed to slow liquid flow and thus coordinate safer swallowing for many patients with oropharyngeal dysphagia. MTLs have commonly been used in skilled nursing facilities, with an average of 8.3% of residents receiving thickened fluids to treat dysphagia in one study of 25,470 residents in a skilled nursing facility [20]. However, there is also evidence in the literature to suggest an increased likelihood of silent aspiration, without overt cough response, with MTLs compared to thin liquids [21]. Miles et al. utilized fiberoptic endoscopy evaluations of swallow to examine safety and cough response with thin and mildly thick liquids in 180 patients with a wide range of medical diagnoses. They found that while patients were more likely to aspirate thin liquid (32%) versus MTLs (18%), those who aspirated MTLs were more likely to do so silently. Hence, MTL diets are not without risk, with various studies in the literature arguing that there is a paucity of quality evidence to justify the current practice of MTL diets [22]. This study seeks to address that paucity by providing data regarding the characteristics and clinical outcomes in hospitalized patients prescribed mildly thick liquids using a stringent algorithm.

Although MTLs are commonly used to manage dysphagia, the impact of MTLs as a temporary bridge to baseline diet has not yet been extensively explored in the tertiary care hospital setting. Unlike in long-term care facilities, the inpatient hospital team is charged with quickly stabilizing a patient, establishing a form of nutrition/medication, and preparing the patient for transfer to the next level of care as soon as medically appropriate. As part of the inpatient team, the hospital-based speech pathologist must evaluate the patient’s risk for pulmonary complications with an oral diet and recommend the least restrictive diet. Often, the alternative to a modified diet such as MTL is a non-oral form of nutrition/hydration. MTLs are frequently considered a temporary alternative to nothing by mouth as they allow an oral route for medication delivery and medical stability for transfer to the next level of care. The alternative to MTLs is often an invasive non-oral feeding tube, which could result in additional complications, prolonged hospitalization, and does not consistently eliminate aspiration risk [23]. Our study seeks to provide a useful evaluation of the utility of mildly thick liquids in a hospital setting by assessing the safety of prescribing mildly thick liquids using an algorithm for patients with acute oropharyngeal dysphagia.

Assessing the risk of aspiration on the mildly thick liquid diet

Most patients who were prescribed MTLs had no signs of aspiration on their radiologic imaging before or after the initial SLP evaluation, which supports the safety of MTLs. Seven patients (4%) were found to have "aspiration" on radiologic imaging after SLP diet recommendation for MTL. Of those seven patients, two patients had imaging taken within too short of a time frame to be likely related to the MTL diet. The remaining patients had confounding medical conditions, making it difficult to determine if the pulmonary outcome was solely due to the MTL diet recommendation.

Equivocal impact of instrumental swallow evaluation

This study also analyzed differences in radiologic findings between patients who underwent an instrumental swallow evaluation versus a bedside swallow evaluation for the follow-up assessment. Fifty patients (30.9%) underwent an instrumental swallow evaluation for follow-up. The results indicate that two patients in this group had concerns for aspiration on radiologic imaging after initiating MTLs. One hundred twelve patients (69.1%) underwent a repeat bedside swallow evaluation for follow-up. Interestingly, the results indicate that there were again only two patients who had concern for aspiration on radiologic imaging in the group that had a repeat bedside swallow follow-up. Based on radiographic findings alone, 45 instrumental evaluations would be needed at follow-up to detect one aspiration event. Thus, the expected clinical impact of instrumental swallow evaluation is quite small compared to skilled observational evaluation. All four of the patients with concern for aspiration in both groups had additional complex medical complications, as documented in Table 2, that likely contributed to the aspiration found on radiologic imaging.

Medically complex patients may routinely be recommended for instrumental evaluation as compared to observational evaluation, which may be a potential confounder behind the equivocal impact of instrumental swallow evaluation. At the same time, given evidence that instrumental swallow evaluations reduce pulmonary complications in patients who are prescribed MTLs, it is pertinent to weigh the risks and benefits of performing instrumental swallow evaluations as a prerequisite for prescribing the MTL diet. The traditional rationale for performing instrumental swallow evaluations hinges upon the assumption that patients may be more likely to aspirate MTL silently. Typically, the instrumental study (videofluoroscopy or endoscopy) would be completed prior to initiating the MTL diet, which means the patient would remain NPO until the instrumental evaluation occurs. It is important to recognize the realities of a hospital setting; ordered instrumental evaluations are challenging to complete same-day, thus forcing the patient to remain without liquids unless they are recommended a modified diet such as mildly thick liquids. Videofluoroscopy and often endoscopy require coordination of multiple disciplines and providers to execute, which may prolong the hospital stay and delay medication delivery. The results in this study indicate that instrumental swallow evaluations resulted in restricting diet recommendations from MTLs in only 4.1% more patients than bedside swallow alone. This presents an opportunity to reevaluate the current practice of performing instrumental studies as a prerequisite for initiation of the MTL diet, a change that would directly impact patient care. Although beyond the scope of this study, a cost-effectiveness analysis may provide additional incentive for advancing to MTL without an instrumental evaluation.

Safety of using mildly thick liquids as a temporary bridge

In recent studies, controversy over the use of MTLs arose due to concern about safety. In animal models, repeated injection of mildly thick liquids into the trachea has been found to result in pulmonary complications [8-9]. In 2018, Nativ-Zeltzer et al. found that rabbits who aspirated 1.5 mL of xanthan gum thickened water had increased pulmonary complications than those who aspirated water alone. Further, aspiration of cornstarch-thickened water was found to be fatal in this animal study. Pulmonary inflammation with one fatality was also found in a group of 19 adult rats who aspirated mildly thick water versus thin water over the course of eight days [9]. While it is unclear if adult humans would have a similar pulmonary response under the same circumstances, it is known anecdotally that aspiration of liquids is more likely to occur in small to trace volumes over the course of a meal, which is quite different from direct injection of liquid into the trachea. Also, the development of aspiration pneumonia in human adults is not solely or directly related to aspiration. Other risk factors for aspiration pneumonia include poor oral hygiene, presence of tube feeding, complexity of medical diagnoses, and dependence for feeding [24]. Also, video fluoroscopic studies of mildly thick liquid safety have identified that post-swallow pharyngeal residue increased aspiration risk in adults6. To address concerns with short-term use of mildly thick liquids, this study assessed the safety of recommending mildly thick liquids at bedside temporarily, while an instrumental study, if it is still required, can be executed once coordinated.

After reviewing the clinical records of 162 patients, we concluded that prescribing mildly thick liquids as a temporary bridge in hospitalized patients via a stringent algorithm is safe, given that the majority did not have a concern for aspiration on subsequent radiologic imaging. Even in the small subset that had possible aspiration noted on imaging after the initial SLP evaluation, further evaluation clarified that many of the patients had confounding underlying medical conditions or changes in status that increased aspiration risk, irrespective of diet status. NPO status has been shown to be associated with poorer survival rates, likely due to a variety of reasons, including more severe dysphagia [25]. As the goal for medical stability in the hospital setting is always to get the patient to a sufficient oral intake (PO) status, mildly thick liquids play an important role as a temporary bridge between no oral diet and normal diet PO. We hope that providing evidence of the very low incidence of aspiration with mildly thick liquids prescribed with bedside swallow evaluation with a stringent algorithm in a study of 162 patients will prompt other clinicians to consider the utility of this modified diet versus nothing by mouth status.

Limitations

Due to the nature of this retrospective study, the authors were unable to study outcomes of the temporary use of mildly thick liquids in those patients who were discharged prior to repeat evaluation. Given the lack of a control group, it is unclear whether a similar group of patients who were recommended to be NPO instead of the MTL diet would have developed pulmonary complications. This single-institution study at a tertiary care hospital with a small number of adverse outcomes suggests mildly thick liquids prescribed after bedside swallow evaluation using a stringent algorithm, which excluded several specific diagnoses, are safe; however, the skilled clinician should always weigh the risks and benefits of a modified diet for each individual patient situation. The authors of this study are also unable to account for variability in SLP bedside swallow evaluation techniques, the acuity of patient diagnoses, and the use of alternate thickening agents, which all may vary across institutions.

The authors were also unable to quantify the total volume of MTL consumed by patients who were prescribed the diet; there may be some patients on the diet who consume more or less than other patients for various reasons (such as access, thirst, mentation, or tolerance of the texture). Although clinicians educate the patient, family, and staff on the need to mix the gel-based thickening product into all drinks, it is unclear whether the instructions are consistently followed, given that the SLPs were unable to supervise all liquid consumption. Finally, although this modified diet likely assisted many patients in discharging to a subacute level of care, these patients were ultimately lost to follow-up, limiting the observations that can be made regarding their outcome.

There is concern for dehydration with long-term use of MTLs. This is due to a dislike of the taste and texture of drinks with thickeners. There may also be reduced access to liquids given the additional time and steps required to thicken a liquid and offer it to a patient20. This study focused on the use of MTLs in hospitalized patients as a temporary bridge to baseline diet and/or to facilitate discharge to the next level of care. Hydration status should always be monitored by clinicians caring for hospitalized patients, especially those without an ad lib diet prescription.

## Conclusions

This study assessed the safety of prescribing MTLs as a temporary bridge to baseline diet in the acute hospital setting. Specifically, a decision-making algorithm was utilized to assess safety of MTLs prescribed at bedside initially without an instrumental evaluation. Despite the use of MTLs as a compensation for dysphagia for many years, data are sparse demonstrating a systematic assessment of safety for temporary use of MTLs in an acute setting. The small number of patients with concern for aspiration after MTL diet was initiated suggests that an instrumental swallow evaluation may not be needed to prescribe MTLs, as long as a sound algorithm is utilized at bedside. Ultimately, these findings indicate MTL diet has a potential benefit to the patient, allowing them to continue an oral diet, and a system-wide benefit, allowing patients to discharge to lower level of care in a potentially shorter timeframe.

## References

[1] Ortega O, Martín A, Clavé P (2017). Diagnosis and management of oropharyngeal dysphagia among older persons, state of the art. J Am Med Dir Assoc.

[2] Rommel N, Hamdy S (2016). Oropharyngeal dysphagia: manifestations and diagnosis. Nat Rev Gastroenterol Hepatol.

[3] Robertson HM, Patillo MS (1993). A strategy for providing food to the patient with neurologically based dysphagia. J Can Diet Assoc.

[4] National Dysphagia Diet Task Force (2002). National Dysphagia Diet: Standardization for Optimal Care.

[5] Bisch EM, Logemann JA, Rademaker AW, Kahrilas PJ, Lazarus CL (1994). Pharyngeal effects of bolus volume, viscosity, and temperature in patients with dysphagia resulting from neurologic impairment and in normal subjects. J Speech Hear Res.

[6] Steele CM, Alsanei WA, Ayanikalath S (2015). The influence of food texture and liquid consistency modification on swallowing physiology and function: a systematic review. Dysphagia.

[7] Maeda K, Koga T, Akagi J (2016). Tentative nil per os leads to poor outcomes in older adults with aspiration pneumonia. Clin Nutr.

[8] Nativ-Zeltzer N, Kuhn MA, Imai DM (2018). The effects of aspirated thickened water on survival and pulmonary injury in a rabbit model. Laryngoscope.

[9] Nativ-Zeltzer N, Ueha R, Nachalon Y (2021). Inflammatory effects of thickened water on the lungs in a murine model of recurrent aspiration. Laryngoscope.

[10] Werden Abrams S, Gandhi P, Namasivayam-MacDonald A (2023). The adverse effects and events of thickened liquid use in adults: a systematic review. Am J Speech Lang Pathol.

[11] Dallal-York J, Segalewitz T, Croft K (2022). Incidence, risk factors, and sequelae of dysphagia mediated aspiration following lung transplantation. J Heart Lung Transplant.

[12] Mukdad L, Toppen W, Nguyen S, Kim K, Mendelsohn AH, Zarrinpar A, Benharash P (2019). A targeted swallow screen for the detection of postoperative dysphagia in liver transplant patients. Prog Transplant.

[13] Reedy EL, Simpson AN, O'Rourke AK, Bonilha HS (2023). Characterizing swallowing impairment in a post-lung transplant population. Am J Speech Lang Pathol.

[14] Dallal York J, Leonard K, Anderson A, DiBiase L, Jeng EI, Plowman EK (2022). Discriminant ability of the 3-ounce water swallow test to detect aspiration in acute postoperative cardiac surgical patients. Dysphagia.

[15] Richmon JD, Feng AL, Yang W, Starmer H, Quon H, Gourin CG (2014). Feasibility of rapid discharge after transoral robotic surgery of the oropharynx. Laryngoscope.

[16] Hutcheson KA, Warneke CL, Yao CM (2019). Dysphagia after primary transoral robotic surgery with neck dissection vs nonsurgical therapy in patients with low- to intermediate-risk oropharyngeal cancer. JAMA Otolaryngol Head Neck Surg.

[17] Langmore SE, Schatz K, Olsen N (1988). Fiberoptic endoscopic examination of swallowing safety: a new procedure. Dysphagia.

[18] Logemann JA (1998). Evaluation and treatment of swallowing disorders. Curr Opin Otolaryngol Head Neck Surg.

[19] Jokerst C, Chung JH, Ackman JB (2018). ACR Appropriateness Criteria(®) acute respiratory illness in immunocompetent patients. J Am Coll Radiol.

[20] Castellanos VH, Butler E, Gluch L, Burke B (2004). Use of thickened liquids in skilled nursing facilities. J Am Diet Assoc.

[21] Miles A, McFarlane M, Scott S, Hunting A (2018). Cough response to aspiration in thin and thick fluids during FEES in hospitalized inpatients. Int J Lang Commun Disord.

[22] O'Keeffe ST (2018). Use of modified diets to prevent aspiration in oropharyngeal dysphagia: is current practice justified?. BMC Geriatr.

[23] Mamun K, Lim J (2005). Role of nasogastric tube in preventing aspiration pneumonia in patients with dysphagia. Singapore Med J.

[24] Langmore SE, Terpenning MS, Schork A, Chen Y, Murray JT, Lopatin D, Loesche WJ (1998). Predictors of aspiration pneumonia: how important is dysphagia?. Dysphagia.

[25] Shune SE, Karnell LH, Karnell MP, Van Daele DJ, Funk GF (2012). Association between severity of dysphagia and survival in patients with head and neck cancer. Head Neck.

